# Glyphosate Toxicity to Native Nontarget Macrophytes Following Three Different Routes of Incidental Exposure

**DOI:** 10.1002/ieam.4350

**Published:** 2020-11-05

**Authors:** Verena Sesin, Christina M Davy, Kevin J Stevens, Rebekah Hamp, Joanna R Freeland

**Affiliations:** ^1^ Environment and Life Sciences, Trent University Peterborough Ontario Canada; ^2^ Wildlife Research and Monitoring Section, Ontario Ministry of Natural Resources and Forestry Peterborough Ontario Canada; ^3^ Department of Biology Trent University Peterborough Ontario Canada; ^4^ Department of Biology Wilfrid Laurier University Waterloo Ontario Canada

**Keywords:** Ecological risk assessment, Mycorrhizae, Scarlet Ammannia, Wetland, Virginia Mallow

## Abstract

A major goal of invasive plant management is the restoration of native biodiversity, but effective methods for invasive plant control can be harmful to native plants. Informed application of control methods is required to reach restoration goals. The herbicide glyphosate, commonly applied in invasive plant management, can be toxic to native macrophytes. Our study assessed the response of 2 macrophytes that are endangered in our study area (*Ammannia robusta* and *Sida hermaphrodita*) to glyphosate concentrations that mimic incidental exposure from nearby invasive plant control: spray drift of 4 × 10^−7^% to 5% glyphosate; pulse and continuous immersion in water containing 2 to 41 µg/L glyphosate; and rhizosphere contact with 5%‐glyphosate‐wicked invasive plants. We assessed macrophyte sensitivity at 14‐d postexposure, and quantified abundance of arbuscular mycorrhizal fungi. Glyphosate spray concentrations as low as 0.1% reduced macrophyte growth. *Ammannia* was more sensitive overall to glyphosate spray than *Sida*, although sensitivity varied among measured endpoints. Conversely, macrophytes were not affected by immersion in low concentrations of glyphosate or rhizosphere contact with a glyphosate‐wicked plant. Likewise, arbuscular mycorrhizal fungi abundance in roots was similar among glyphosate‐sprayed and control plants. Based on our results, we recommend that invasive plant managers reduce risks to native nontarget plants through implementing measures that limit off‐target spray drift, and consider the feasibility of more targeted applications, such as with wick equipment. *Integr Environ Assess Manag* 2021;17:597–613. © 2020 The Authors. *Integrated Environmental Assessment and Management* published by Wiley Periodicals LLC on behalf of Society of Environmental Toxicology & Chemistry (SETAC)

## KEY POINTS

- We assessed the response of nontarget, endangered macrophytes and associated mycorrhizal fungi to glyphosate exposures that mimicked realistic scenarios encountered during invasive plant control.
- Glyphosate toxicity to macrophytes varied with the exposure pathway, where spray at low concentrations reduced native macrophyte growth, but no effects were detected from low‐level surface water residues or adjacent wick‐application.
- Mycorrhizal fungi in macrophyte roots were not affected by glyphosate spray.
- We recommend that glyphosate‐based invasive plant management implements measures to limit off‐target spray drift, and that risk assessment procedures for nontarget macrophytes consider adding spray exposure tests with emergent species to their repertoire.

## INTRODUCTION

Over the past 3 decades, alien invasive plants have been recognized as a serious threat to native plant biodiversity (Vitousek et al. 1997; D'Antonio and Meyerson 2002; Zedler and Kercher 2004). While native plants typically occur in diverse plant communities and provide habitat for many other biota (Chambers et al. 2008; Rejmankova 2011), invasive plants often form monocultures that exclude many species, thereby reducing biodiversity and altering habitat structure (Zedler and Kercher 2004; Rejmankova 2011). In addition, invasive plants can impact ecosystem functioning by disrupting food chains, and altering nutrient cycling and water quality (Pimentel et al. 2005; Rejmankova 2011; Pyšek et al. 2012). Thus, control of invasive plants with the goal of protecting and restoring native plant biodiversity is a frequent activity of restoration practitioners (D'Antonio and Meyerson 2002; Kettenring and Adams 2011).

Application of herbicides is a common control method for invasive plants (Kettenring and Adams 2011). Glyphosate is the most widely used herbicide for controlling invasive plants in agricultural lands, urban environments, and wildlands including habitats such as forests, wetlands, rangelands, roadsides, and power line corridors (Kettenring and Adams 2011; Benbrook  2016). Globally, glyphosate use in agriculture has risen almost 15‐fold since the late 1990s following the introduction of glyphosate‐resistant crops; additionally, nonagricultural uses have increased 5‐fold over the same timeframe (Benbrook 2016). For example, from 2007 to 2011, glyphosate was sprayed on approximately 300 000 ha of public wildlands in the United States (Wagner et al. 2017). Glyphosate is so widely used in part because it is nonselective and systemic: It inhibits the 5‐enolpyruvylshikimate 3‐phosphate synthase in the shikimate pathway, a pathway that is essential for the synthesis of aromatic amino acids in all plants (Herrmann and Weaver 1999; Baylis 2000; Pérez et al. 2011). Moreover, glyphosate affects both aboveground shoots and belowground roots and rhizomes of treated plants, because it is phloem‐mobile and readily translocated throughout the plant (Pérez et al. 2011). While these properties make glyphosate an effective control agent for invasive plants, its nonselectivity may also threaten nontarget native species that grow alongside treated invasive plants (Gardner and Grue 1996; Gettys and Sutton 2004).

Glyphosate is applied to invasive plants through various methods, including foliar spray, stem injection, and wiping or wicking of the leaves (Evans 1982; Carlisle and Trevors 1988; Kay 1995; Krueger‐Mangold et al. 2002; Solomon and Thompson 2003; Wagner et al. 2017; Quirion et al. 2018). When glyphosate is applied on target invasive plants, it may also expose nearby nontarget, native plants through several pathways, including spray drift towards nontarget plants following foliar spray application on target plants (Payne et al. 1990; Reddy et al. 2010; Mateos‐Naranjo and Perez‐Martin 2013). Glyphosate can also enter waterbodies by spray drift and runoff following applications in adjacent agricultural fields, wildlands, or urban areas, and can be present in surface water, groundwater, soil, and sediments at low levels (Scribner et al. 2007; Peruzzo et al. 2008; Struger et al. 2008; Tsui and Chu 2008; Ruiz‐Toledo et al. 2014). Moreover, treated plants may transfer glyphosate to the rhizosphere, which could also affect adjacent native plants (Coupland and Lutman 1982; Neumann et al. 2006; Tesfamariam et al. 2009). If 1 goal of invasive plant control activities is to protect and restore native vegetation, managers should understand whether application techniques are leading to exposure to residual glyphosate concentrations and if this exposure can negatively impact native plants and thereby hinder successful restoration.

The effects of residual glyphosate on nontarget plants have been well studied for species of terrestrial ecosystems such as agricultural field edges, grasslands, or forests (Coupland and Lutman 1982; Marrs et al. 1993; Power et al. 2013; Boutin et al. 2014; Damgaard et al. 2014; Aguilar‐Dorantes et al. 2015; Florencia et al. 2017). From these studies it is apparent that glyphosate can cause phytotoxic effects in nontarget plants, although glyphosate sensitivity can vary among species and also among life stages of a species. However, results of these studies may not be directly transferrable to invasive plant control activities in aquatic and semiaquatic ecosystems due to differences in glyphosate application rates, required buffer zones, and the biologies of nontarget plant species. Only a few studies have looked at the responses of nontarget aquatic and semiaquatic plants (macrophytes) to unintended glyphosate exposures from invasive plant control activities (Kay 1995; Gardner and Grue 1996; Gettys and Sutton 2004; Brisson et al. 2006; Mateos‐Naranjo and Perez‐Martin 2013). These studies recorded damage from adjacent glyphosate‐based invasive plant control to nontarget macrophyte species, whether exposure resulted from adjacent foliar spray (Kay 1995; Gardner and Grue 1996; Gettys and Sutton 2004; Brisson et al. 2006), wiping or wicking (Kay 1995), or water residues (Mateos‐Naranjo and Perez‐Martin 2013). These studies have contributed to our knowledge of the potential impacts on nontarget macrophytes from invasive plant control activities in aquatic and semiaquatic ecosystems; however, observations are in several cases anecdotal (e.g., Kay 1995; Brisson et al. 2006), and it remains difficult to draw conclusions about the range of responses of nontarget macrophytes to glyphosate applied through different methods on adjacent invasive plants.

Predicting the impacts of unintentionally exposing plants to glyphosate is further complicated by the fact that responses to glyphosate are not independent of the environment in which they are growing. Arbuscular mycorrhizal fungi (AMF) are symbiotic fungi in the soil that form associations with plant roots (Smith and Read 2008). The AMF associations occur in about 80% of plant species (Strack et al. 2003) including rooted wetland macrophytes (Kandalepas et al. 2010; Stevens et al. 2010, 2011). The AMF affect plant growth and development by supporting nutrient and water acquisition (Smith and Read 2008), and mediating effects of contaminants (Twanabasu et al. 2013). They can also influence plant herbicide tolerance (Ronco et al. 2008; Lekberg et al. 2017). Despite the abundance of AMF, and the existence of several studies that have reported interactive effects between glyphosate and AMF in in vitro and soil exposure tests (Estok et al. 1989; Chakravarty and Chatarpaul 1990; Ronco et al. 2008), the effects on in vivo AMF associated with nontarget macrophytes exposed to glyphosate have not been quantified. This is a potentially important part of understanding unintentional glyphosate exposure because a reduced abundance of AMF could affect plant health.

The goal of the present paper was to assess the sensitivity of nontarget, native macrophytes and their associated AMF following incidental glyphosate exposure. We evaluated 3 “realistic” pathways mimicking unintended exposure: Our first pathway was exposure to glyphosate spray drifting from nearby foliar applications on invasive macrophytes. Foliar application of glyphosate is commonly used to control invasive macrophytes in aquatic and semiaquatic habitats, such as *Phragmites* spp., *Typha* spp., and *Scirpus* spp. (Solberg and Higgins 1993; Ailstock et al. 2001). Our second pathway was exposure to pulse and continuous glyphosate residues in surface water that may result from nearby applications. Glyphosate has been detected year‐round in surface water grab samples taken in a bay which receives input from both invasive plant control applications and agricultural applications (CM Davy, Ontario Ministry of Natural Resources and Forestry, Peterborough, ON, Canada, personal communication). Our third pathway was exposure via rhizosphere contact with glyphosate‐wicked invasive macrophytes, which could transfer glyphosate via their roots and rhizomes (Coupland and Lutman 1982). We quantified sensitivity based on an array of endpoints, with the goal of identifying toxicologically sensitive and ecologically relevant endpoints (Arts et al. 2008; Maltby et al. 2010) that can also be used in future studies investigating the sensitivity of nontarget macrophytes to herbicides.

We selected 2 emergent nontarget macrophyte species for our study, *Ammannia robusta* Heer & Regel (Scarlet Ammannia; hereafter *Ammannia*) and *Sida hermaphrodita* (L.) Rusby (Virginia Mallow; hereafter *Sida*), which are both native to Ontario, Canada, and form associations with AMF (Stevens et al. 2010; Mulholland 2019). *Ammannia* is an annual plant up to 1 m tall with opposite leaves and unstalked, pale lavender flowers in the leaf axils (Douglas and Oldham 1999). *Sida* is an herbaceous perennial up to 3 m tall with alternate leaves along the stem, and white flowers in stalked clusters at the upper leaf axils (Spooner et al. 1985). Both species are endangered in Canada, with only a few extant populations. Two small *Sida* populations are known in Ontario, 1 growing in a marsh dominated by *Typha latifolia* L. and *Phragmites australis* (Cav.) Trin. ex Steud., and another along a roadside and pipeline corridor; both of these sites are periodically flooded (COSEWIC 2010). *Ammannia* has 2 known extant populations in British Columbia, and 4 in Ontario, all of which grow in open, shoreline, and semiaquatic habitats alongside invasive macrophytes such as *P. australis* or *Lysimachia nummularia* L. (Environment Canada 2015a). Government mandated recovery strategies for both endangered species include invasive plant control, for which best management practices are developed (Environment Canada 2015a, 2015b). For the frequently cooccurring invasive *P. australis*, glyphosate application is commonly used as effective control practice, with spray application recommended for dense monoculture stands, and wicking for small stands or individual plants (OMNR 2011). Thus, both *Ammannia* and *Sida* could be exposed to glyphosate residues from invasive plant control activities. Understanding potential effects of glyphosate residues on nontarget, endangered macrophytes can both inform effective restoration strategies for native plant communities in invaded aquatic and semiaquatic habitats, and contribute to the development of sustainable management practices for invasive plants.

## MATERIALS AND METHODS

### Overview of performed experiments

We performed a total of 4 experiments. To evaluate the sensitivity of nontarget macrophytes and their associated AMF to glyphosate spray drift, we performed 2 separate experiments, exposing either *Ammannia* (experiment 1) or *Sida* (experiment 2) to a range of glyphosate spray concentrations. The response of nontarget macrophytes to glyphosate residues in surface water was assessed based on an experiment with *Ammannia* exposed to low‐level pulse and continuous glyphosate concentrations (experiment 3). Potential effects on nontarget macrophytes from exposure via rhizosphere contact with glyphosate‐wicked invasive macrophytes were evaluated by assessing the response of *Ammannia* plants grown adjacent to glyphosate‐wicked *T. latifolia* plants (hereafter *Typha*, representing an invasively growing wetland plant) (experiment 4).

#### Glyphosate

We used a formulated product of glyphosate for the present paper: Roundup WeatherMAX with Transorb 2 Technology Liquid Herbicide (540 g a.i./L; Monsanto Canada, Winnipeg, MB, Canada). Roundup WeatherMAX is registered for the control of invasive macrophytes (e.g., *Phragmites* and *Typha*) in locations such as roadside ditches. Roundup WeatherMAX contains 48.8% active ingredient glyphosate as potassium salt (potassium *N*‐[(hydroxyphosphinato)methyl]glycine). The remaining 51.2% includes surfactant, water, and other minor ingredients (Monsanto Canada Inc. 2018), the specifics of which were not disclosed by the manufacturer upon request (Monsanto Canada Technical Support, Winnipeg, MB, Canada, personal communication).

#### Macrophyte propagation

All plants were grown from seeds under greenhouse conditions, allowing us to use plants of known age and without previous exposure to glyphosate. We collected *Ammannia* seeds from greenhouse grown plants that were established from soil cores collected from natural populations on Pelee Island, Ontario, Canada. Soil cores were obtained in 2017 and seeds collected in 2018 (ESA 17(2)(b) permit AY‐B‐009‐17). To obtain the soil sample, a split‐core sampler with an auger tip (AMS, American Falls, ID, USA) was used and collected soil stored in a plastic bag. Soil cores were brought to the greenhouse at Wilfrid Laurier University, Waterloo, Ontario, Canada, and hydrated with deionized water. Hydrated samples were homogenized by hand to disperse soil clumps, and all nonsoil debris was removed. The homogenized soil cores were evenly distributed across the surface of a 50:50 mix by volume of Premium Pro‐mix Mycorrhizae Pro soil mixture (Premier Tech Horticulture, Quakertown, PA, USA) and nepheline syenite (Unimin Canada Ltd, Havelock, ON, Canada) in plastic seedling trays (52 × 26 × 6 cm) with clear plastic lids. Soil was kept moist throughout the experiment and maintained under greenhouse conditions (16:8 light:dark cycle, ~24–30 °C) for 6 m. Seed capsules were removed from mature *Ammannia* plants, dried at room temperature, and seeds were collected and stored dry at 4 °C. *Sida* seeds were collected from mature plants found in a natural population in Haldimand County, Ontario, Canada, in fall, 2013. Seeds were collected in paper bags and stored dry at 4 °C. Exact locations for both species cannot be disclosed due to their protected status in Ontario.

In September and October 2017, *Typha* inflorescences were cut from plants growing in a roadside ditch in Peterborough, Ontario, Canada (latitude: 44.335; longitude: −78.282). Inflorescences were dried at room temperature, and dry seeds were removed from the stalks and transferred to sealed plastic bags. *Typha* seeds were stored at 4 °C until use.

*Ammannia* and *Typha* plants were grown in a greenhouse at Trent University, Peterborough, Ontario, Canada, whereas *Sida* plants were grown in a greenhouse at Wilfrid Laurier University, Waterloo, Ontario, Canada. In February 2019, we prepared *Typha* seeds following the methods of Ahee et al. (2015), and placed them in a sterile Petri dish half‐filled with deionized water in a greenhouse to germinate. *Typha* seedlings were grown following the methods of Freeland et al. (2020). In May 2019, *Ammannia* seeds were placed on premoistened soil (Sunshine professional growing mix #15; Sungro, Agawam, MA, USA) filled in 200‐cell plug trays (1 seed per cell), and maintained in the same way as the *Typha*. In July 2019, *Sida* seeds were soaked in water for 15 min and scarified by puncturing the convex side with a needle to increase germination rates (Spooner et al. 1985). Seeds were then placed on premoistened soil (one‐half natural soil collected from an area supporting a natural population of *Sida* in Haldimand County, Ontario, Canada; one‐quarter White Lightning nepheline syenite sand, Smelko Foundry Products Ltd, Milton, Ontario, Canada; and one‐quarter Premier Pro‐mix Mycorrhizae Pro soil mixture, Premier Tech Horticulture, Quakertown, PA, USA) in 36‐cell plug trays (3 seeds per cell to account for low germination success), which were placed in flats with openings for drainage, and covered with clear plastic domes to maintain humidity. Plants were watered daily.

### Experiment 1: *Ammannia* exposed to foliar glyphosate spray

#### Experimental design

The goal of this experiment was to assess the sensitivity of *Ammannia* exposed to foliar glyphosate spray. Our experimental design consisted of 6 glyphosate treatments, which were concentrations of 5, 1.34, 0.36, 0.1, 7.56 × 10^−6^, and 3.52 × 10^−7^%, and a 0% control treatment (glyphosate‐free municipal tapwater; see Table 1 for rationale and Supplemental Data Table S1 for respective measured concentrations). Methylated seed oil was added at 1% v/v to 5, 1.34, 0.36, and 0.1% glyphosate treatments (concentrations used for experiments 1 and 2 only; Table 1) to increase wetting and thereby facilitate penetration into plant tissues, which is common practice for invasive plant spray applications in Ontario (Howell 2017; Tozer and Mackenzie 2019). Glyphosate treatments with and without methylated seed oil were not statistically compared with each other. Treatment concentrations are hereafter expressed in spray rates (%), as these are most relevant to the hand‐held and backpack spray operations that we mimic in the present paper (Howell 2017; Monsanto Canada ULC 2019). For each of the 7 treatments, we had 6 replicates (i.e., 6 different plants were sprayed), resulting in a setup with a total of 42 *Ammannia* plants.

**Table 1:** Rationale for applied nominal glyphosate treatment concentrations in the experiments assessing glyphosate effects from foliar spray exposure to *Ammannia* (experiment 1) and *Sida* (experiment 2), from surface water residues exposure to *Ammannia* (experiment 3), and from rhizosphere contact of *Ammannia* with an adjacent glyphosate‐wicked invasive plant (experiment 4)

Glyphosate treatment (%)	Glyphosate treatment (g/L)	MSO surfactant added at 1% v/v	Experiment	Rationale
5.00	27.0	Yes	1, 2, 4	Rate used to control invasive *Phragmites* in some Ontario wetlands (Howell 2017; Tozer and Mackenzie 2019). Imitates realistic spray drift or adjacent wicking exposure on nontarget plants
1.34	7.2	Yes	1, 2	Rate advised for control of several perennial weeds and brush using hand‐held spray, wick or wipe equipment (Monsanto Canada ULC 2019). Imitates realistic spray drift on nontarget plants adjacent to target spray area
0.36	1.9	Yes	1, 2	Lower concentration that may imitate spray drift on nontarget plants adjacent to target spray area. Calculated as part of a geometric series with factor of 3.73 determined by dividing 5% by 1.34%
0.10	0.5	Yes	1, 2	Lower concentration that may imitate spray drift on nontarget plants adjacent to target spray area. Calculated as part of a geometric series with factor of 3.73 determined by dividing 5% by 1.34%
7.56 × 10^−6^	40.8 × 10^−6^	No	1, 2, 3	Maximum concentration of glyphosate measured in Southern Ontario, Canada, surface waters sampled by Struger et al. (2008). Imitates glyphosate concentrations that nontarget plants could be exposed to in water. Used in foliar spray experiment for comparison
3.52 × 10^−7^	1.9 × 10^−6^	No	1, 2, 3	Maximum concentration of glyphosate measured in Rondeau Bay, Ontario, Canada, surface waters (CM Davy, Ontario Ministry of Natural Resources and Forestry, personal communication). Imitates glyphosate concentrations nontarget plants could be exposed to in water. Used in foliar spray experiment for comparison
0	0	No	1, 2, 3, 4	Control treatment: Glyphosate‐free municipal tapwater

MSO = methylated seed oil.

#### Experimental procedure

In June 2019, 42 *Ammannia* plants at 5‐wk age were transplanted from plug trays into round plastic pots (diameter: 10.2 cm, height: 9.0 cm). All *Ammannia* pots were placed into individual round plastic trays (diameter: 15.0 cm, height: 5.5 cm) filled with tapwater to 1 cm below tray rim (~340 mL). We arranged the 42 *Ammannia* plants in a randomized 4 × 18 block (note: the 42 pots were mixed randomly with the 30 identical pots of experiment 3) on a bench in the greenhouse at Trent University, Peterborough, Ontario, Canada.

To initiate the experiment, on 10 July 2019, plants were moved outside the greenhouse and individually surrounded with cardboard, to avoid potential spray drift on nearby plants and exposure of the greenhouse facilities to glyphosate. Each plant was dosed with 2.2 mL of 1 of the 7 treatments described above, by evenly spraying each plant using motorized hand‐gun equipment (Instapark AHS‐803 Rechargeable Electronic Water Mist Sprayer). Initial trials determined a volume of 2.2 mL as sufficient to fully wet the plants but without runoff. Based on 2.2 mL volume of spray solution and area of each plant tray (1.77 × 10^−6^ ha), our applied glyphosate treatments of 3.52 × 10^−7^% to 5% translated into application rates of 2.37 × 10^−6^ to 33.60 kg glyphosate/ha. Plants were moved back inside the greenhouse once the leaves had fully dried (~0.5–2 h postspray). Plants were monitored and watered daily for a total of 14 d, until 24 July 2019, because first symptoms of glyphosate exposure may be visible only after 7 to 10 d (Baylis 2000; Monsanto Canada ULC 2019). During the 14‐d experimental period, the greenhouse air temperature was on average (±standard error) 28.7 ± 0.2 °C and natural light intensity was 19 863.9 ± 932.9 lx.

#### Experimental assessments

We assessed *Ammannia* response to glyphosate spray by measuring 6 growth‐related endpoints: Total shoot length (combined length of main shoot and all side shoots), main shoot length, number of side shoots, total shoot dry weight, total shoot dry matter content, and total root dry weight. For details on how each endpoint was measured, please see Supplemental Data Text S1.

To confirm applied glyphosate spray concentrations, we submitted 150 mL of each of our 7 treatment solutions to the Agriculture and Food Laboratory (AFL) at University of Guelph (Guelph, ON, Canada) for analysis of glyphosate. To quantify how much glyphosate and aminomethylphosphonic acid (AMPA; 1 common degradation product) is present in the rhizosphere surrounding *Ammannia* at 14 d postspray, we collected and submitted an approximately 20‐g soil sample from 4 replicates for each treatment to AFL. Details on the glyphosate analysis at AFL can be found in Supplemental Data Text S3.

### Experiment 2: *Sida* exposed to foliar glyphosate spray

#### Experimental design

The goal of this experiment was to assess the sensitivity of *Sida* and associated AMF exposed to foliar glyphosate spray. Our experimental design was identical to the design for *Ammannia* described in the *Experimental procedure* section. In brief, this experiment included 6 different glyphosate treatments and 1 control treatment, each with 6 replicates, resulting in a setup with a total of 42 *Sida* plants.

#### Experimental procedure

In August 2019, 42 *Sida* individuals at 4‐wk age were transplanted from plug trays into round plastic pots (diameter: 15.0 cm, height: 13.0 cm) containing Premier Pro‐mix Mycorrhizae Pro soil mixture (Premier Tech Horticulture, Quakertown, PA, USA). All *Sida* pots were placed into individual round plastic trays (diameter: 15.0 cm, height: 5.5 cm) filled with tapwater to 1 cm below tray rim (~250 mL). We arranged the 42 *Sida* plants in a randomized 6 × 7 block on a bench in the greenhouse at Wilfrid Laurier University, Waterloo, Ontario, Canada.

The experiment was initiated on 29 August 2019 following the same procedure as for *Ammannia* described in the *Experimental assessments* section. Plants were monitored and watered daily for a total of 14 d, until 13 September 2019. During the 14‐d experimental period, the greenhouse at Wilfrid Laurier University was programmed to maintain a temperature cycle of 16 h at 21 °C and 8 h at 17 °C, with artificial lights turning on between 6:00 and 22:00 if outdoor lighting was below 50 W/m^2^.

#### Experimental assessments

We assessed *Sida* response to glyphosate spray by measuring 6 growth‐related endpoints: main shoot length, total surface area (leaves and stem), proportion of healthy surface area, total shoot dry weight, total shoot dry matter content, and total root dry weight. For details on how each endpoint was measured, please see Supplemental Data Text S1.

We analyzed mycorrhizal colonization in *Sida* roots as the proportion of root cortical cells colonized by the 3 main AMF structures: arbuscules, vesicles, and intraradical hyphae. Subsamples of harvested *Sida* roots from all glyphosate spray treatments were rinsed in tapwater and then fixed and stored in 50% ethanol. A modification of the ink and vinegar staining technique (Vierheilig et al. 1998) was utilized for visualization of AMF structures (Mulholland 2019), and colonization levels were assessed using the magnified intersections method (McGonigle et al. 1990). Details are given in Supplemental Data Text S2.

To confirm applied glyphosate spray concentrations, we submitted 150 mL of each of our 7 treatment solutions to AFL for analysis of glyphosate. To quantify how much glyphosate and AMPA is present in the rhizosphere surrounding *Sida* at 14‐d postspray, we collected and submitted an approximately 20‐g soil sample from 4 replicates for each treatment to AFL. Details on the glyphosate analysis at AFL can be found in Supplemental Data Text S3.

### Experiment 3: *Ammannia* exposed to glyphosate in surface water

#### Experimental design

The goal of this experiment was to assess the sensitivity of *Ammannia* exposed to pulse and continuous glyphosate concentrations in their surroundings, thereby mimicking exposure to glyphosate residues in surface water. Our experimental design consisted of 5 treatments: 2 single pulse exposures of either 40.8 or 1.9 µg/L glyphosate, 2 continuous exposures of either 40.8 or 1.9 µg/L glyphosate, and a 0 µg/L glyphosate (tapwater) control treatment (see Table 1 for rationale, and Supplemental Data Table S1 for analytical confirmation of concentrations). Each treatment had 6 replicates (i.e., 6 different plants), for a total of 30 *Ammannia* plants. Unfortunately, 1 replicate of the control treatment was knocked over in the greenhouse, leaving us with 5 instead of 6 replicates for the control for all further analysis.

#### Experimental procedure

In June 2019, 30 *Ammannia* plants at 5‐wk age were transplanted from plug trays into round plastic pots (diameter: 10.2 cm, height: 9.0 cm) that were placed into individual round plastic trays (diameter: 15.0 cm, height: 5.5 cm) filled with tapwater to 1 cm below the tray rim (~340 mL). We arranged the 30 *Ammannia* plants in a randomized 4 × 18 block (note: the 30 pots were mixed randomly with the 42 identical pots of experiment 1) on a bench in the greenhouse at Trent University, Peterborough, Ontario, Canada.

To initiate the experiment on 10 July 2019, we replaced the water in each of the 30 trays (each containing 1 *Ammannia* plant) with 340 mL of 1 of the 5 treatments. For a total of 14 d until 24 July 2019, plants were monitored, and trays were topped up daily, using tapwater for the control and pulse exposure trays (so that glyphosate exposure was not renewed, representing a pulse exposure). Trays of the 2 continuous exposure treatments were topped up with their respective glyphosate solutions (so that glyphosate exposure was continuous for 14 d). During the 14‐d experimental period, the greenhouse air temperature averaged (±standard error) 28.7 ± 0.2 °C and natural light intensity was 19 863.9 ± 932.9 lx.

#### Experimental assessments

We assessed *Ammannia* responses by measuring 6 growth‐related endpoints, as described in the *Experimental design* section. In order to quantify glyphosate uptake from the water into plant aboveground tissues, we placed all fresh *Ammannia* shoots in individual plastic bags, immediately froze them at −20 °C, and sent them on ice to AFL for analysis of glyphosate and AMPA residues in plant tissues. To confirm glyphosate treatment concentrations, we submitted 150 mL of each of our mixed treatment solutions to AFL for analysis of glyphosate. As it was necessary to prepare second batches for our 2 continuous glyphosate treatment solutions, we also submitted 150 mL of these batches to AFL for glyphosate analysis. Moreover, we submitted a 150‐mL sample collected from each of 3 replicates of the 2 continuous exposure treatments on day 14 (24 h after the last top‐up) to quantify glyphosate concentrations in the continuous exposure treatments. Details on the glyphosate analysis at AFL can be found in Supplemental Data Text S3.

### Experiment 4: *Ammannia* growing next to a glyphosate‐wicked invasive plant

#### Experimental design

The goal of this experiment was to assess the sensitivity of *Ammannia* exposed to glyphosate via rhizosphere contact with glyphosate‐wicked *Typha*. Our experimental design included 3 treatments: a glyphosate treatment (“W‐5%”) for which we wicked the *Typha* growing next to the *Ammannia* with a 5% solution (Table 1); 1 control treatment (“W‐0%”) testing for potential effects of wicking, in which we wicked the *Typha* growing next to the *Ammannia* with tapwater; and a second control treatment (“NC”) accounting for potential allelopathic effects from *Typha* root extracts (Gallardo et al. 1998), in which *Ammannia* was grown singly (i.e., in a *Typha*‐free pot). For each of the 3 treatments, we had 6 replicates (i.e., 6 different plants were exposed) for a total of 18 *Ammannia* plants.

#### Experimental procedure

In June 2019, 18 *Ammannia* plants at 5‐wk age were transplanted from plug trays into round plastic pots (diameter: 15.2 cm, height: 14.0 cm). Into 12 of the 18 large pots (used for the W‐5% and W‐0% treatments), we also transplanted 1 *Typha* plant so that the *Ammannia* and *Typha* in each pot were spaced approximately 6 cm apart (see Supplemental Data Figure S1). We acknowledge that the treatments vary in plant density (1 plant in the NC treatment versus 2 plants in the W‐5% and W‐0% treatments); however, density‐related effects such as resource competition are likely to be minimal in a 14‐d experimental period. All *Ammannia* pots were placed into individual round plastic trays (diameter: 15.0 cm, height: 5.5 cm) filled with tapwater to 1 cm below tray rim (~150 mL). We arranged the 18 *Ammannia* pots in a randomized 3 × 6 block on a bench in the greenhouse at Trent University, Peterborough, Ontario, Canada.

To initiate the experiment, on 10 July 2019, plants were moved outside the greenhouse. For pots that contained a *Typha* plant (W‐5% and W‐0% treatments), we wicked the *Typha* from soil level to leaf tips with a hand‐held wash mitt (Simoniz Microfibre Wash Mitt, Product #39‐7025‐0; Canadian Tire, Toronto, Canada) previously wetted with 22 mL either 5% glyphosate or tapwater depending on the treatment. During glyphosate wick‐applications on *Typha*, *Ammannia* plants in all pots were covered with an individual plastic bag to ensure that no glyphosate would directly touch the *Ammannia*. The Roundup WeatherMAX label changed in summer 2019, and the new label states that glyphosate is not to be applied using hand‐wicking (Monsanto Canada ULC 2019); however, we assume that glyphosate exposure from our hand‐wicking is similar to exposure using other wick devices as allowed by the label, such as roller or wiper applicators that apply glyphosate by rubbing the invasive plant with an absorbent material containing the glyphosate solution. The experiment ran for 14 d until 24 July 2019, during which time plants were monitored and watered daily. The greenhouse air temperature averaged (±standard error) 28.7 ± 0.2 °C and natural light intensity was 19 863.9 ± 932.9 lx.

#### Experimental assessments

We assessed *Ammannia* response to glyphosate‐wicking of the adjacent *Typha* plant by measuring 4 growth‐related endpoints: total shoot length (combined length of main shoot and all side shoots), main shoot length, number of side shoots, and total shoot dry weight. For details on how each endpoint was measured, see Supplemental Data Text S1. To confirm the glyphosate treatment concentration, we submitted 150 mL of our mixed treatment solution to AFL for analysis of glyphosate. Details on the glyphosate analysis at AFL can be found in Supplemental Data Text S3.

### Statistical analyses

Statistical analyses were conducted with R (R Core Team 2018). For the experimental endpoints “total shoot length,” “main shoot length,” and “number of side shoots,” we calculated the average specific growth rates over the 14‐d experimental period as outlined in OECD (2014) which account for the initial size of each individual plant; growth rates for these endpoints are hereafter stated as *r*_*TSL*_ (14‐d growth rate based on total shoot length), *r*_*MSL*_ (14‐d growth rate based on main shoot length), and *r*_*SS*_ (14‐d growth rate based on number of side shoots).

We analyzed all assessment endpoints with one‐way analysis of variance (ANOVA; “aov” function in package “stats”) and evaluated the models with statistical and visual diagnostics tools for model fit, normality of residuals, and variance homogeneity (ECCC 2007). If assumptions were met, we compared treatment effects to the control using Dunnett's multiple comparisons test (“glht” function in package “multcomp,” specifying “Dunnett” as linear hypotheses). If none of the assumptions were met, we reanalyzed the data with Kruskal‐Wallis rank sum test (“kruskal.test” function in package “stats”), followed by Dunn's pairwise test for multiple comparisons of mean rank sums with 1 control (“dunn.test.control” function in package “PMCMR”). We considered results to be significantly different at *p* < 0.05 (Dushoff et al. 2019).

Foliar spray data (experiments 1 and 2) were analyzed using the package “drc” (Ritz and Streibig 2016) to fit concentration‐response curves and estimate the median EC50 (concentration estimated to cause an effect to 50% of test plants) for each assessment endpoint based on the curves and their associated confidence bounds. For modeling, we considered the glyphosate treatments ranging from 0.1% to 5% (all containing methylated seed oil) and the control treatment. The 2 lower glyphosate spray treatments were not included in concentration‐response modeling, because as noted earlier we did not compare glyphosate treatments with and without methylated seed oil added. For each macrophyte species, we tested a selection of concentration‐response models using the “mselect” function, which compares models using the following criteria: log likelihood value, Akaike's information criterion (AIC), estimated residual standard error, and lack‐of‐fit test *p*‐value (Ritz et al. 2015). We chose the “best‐fitting” model for absolute EC50 and confidence interval estimation via the “ED” function, and evaluated it using statistical and visual diagnostics tools for model fit, normality of residuals, and variance homogeneity (Stephenson et al. 2000; ECCC 2007). All model assumptions were met on untransformed data. Fitted models for each assessment endpoint for *Ammannia* and *Sida* are listed in Supplemental Data Table S2. For concentration‐response curves modeled with the Brain‐Cousens function (Supplemental Data Table S2), which is a modified log‐logistic model for describing hormesis, maximum hormesis (% of control; assuming the control is 100%) was quantified with the “ED” function.

## RESULTS

### Experiment 1: *Ammannia* exposed to foliar glyphosate spray

**Figure 1:**
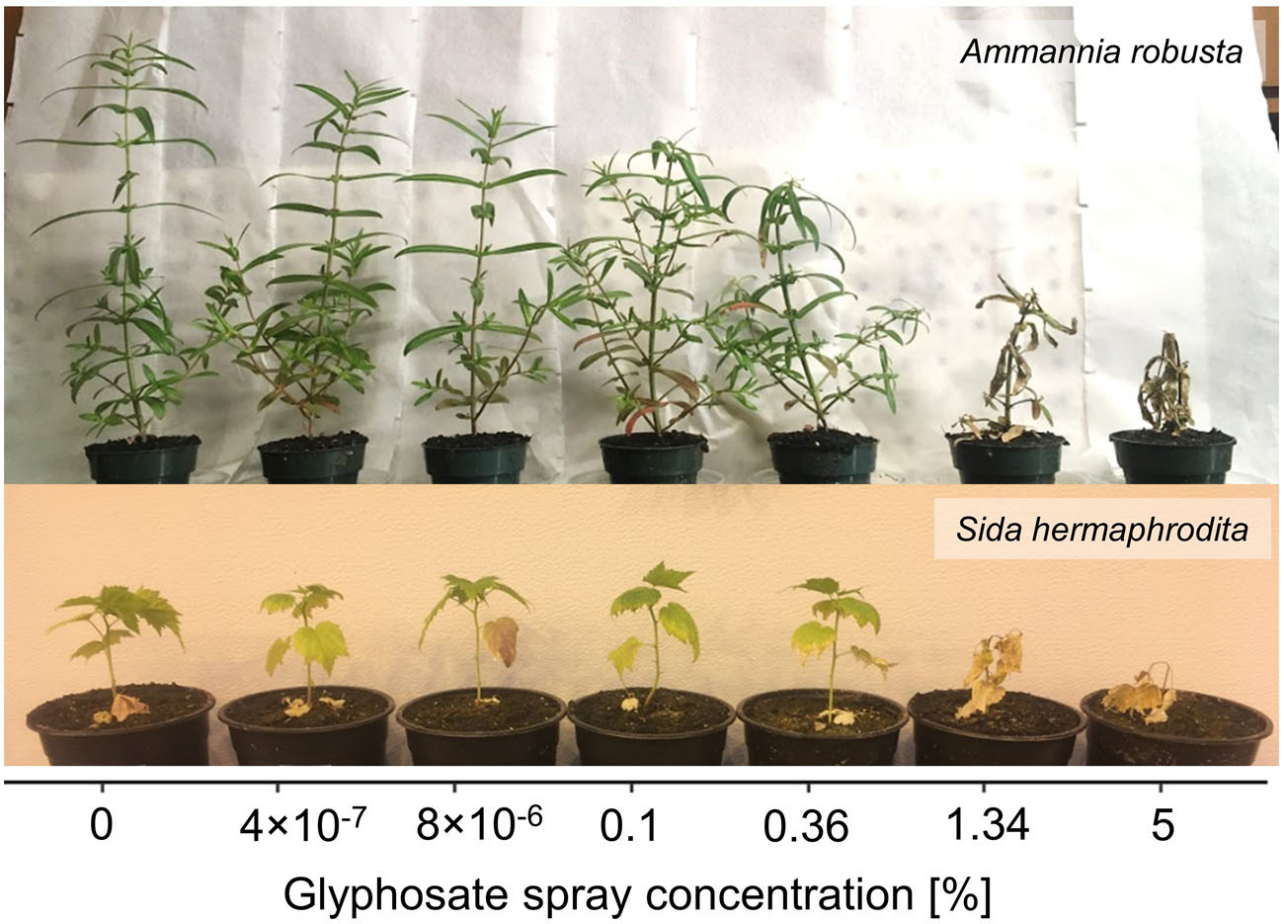
Representative replicates of the control treatment (0%) and each of 6 glyphosate treatments, 4 × 10^−7^% to 5% (2 × 10^−6^–27 g/L; see Supplemental Data Table S1 for measured concentrations) at 14‐d postexposure to spray application for both *Ammannia robusta* and *Sida hermaphrodita* (experiments 1 and 2). Plants show visual symptoms that range from wilting and yellowing of the leaves to complete browning and deterioration and death of tissues.

**Figure 2:**
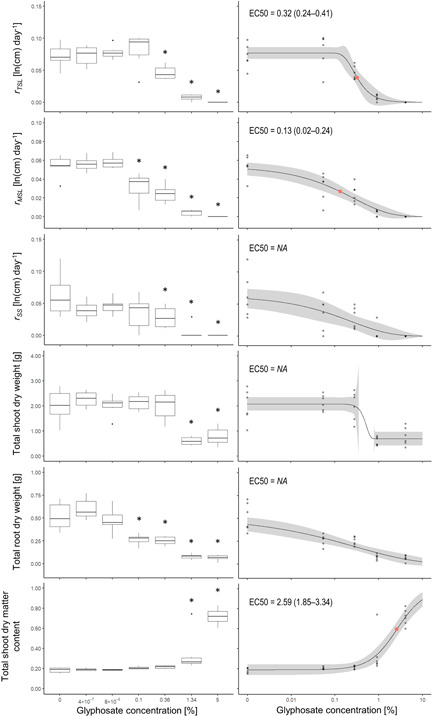
*Ammannia robusta* response to 0% to 5% (0–27 g/L) glyphosate spray 14‐d postexposure assessed based on 6 different endpoints (experiment 1). Each treatment was replicated 6 times. The left boxplot panels show results of an ANOVA analysis with Dunnett's posthoc test where “*” indicates a significant difference of a treatment to the control. The right panels show concentration‐response relationships for the same growth endpoints; measured concentrations were used for modeling (Supplemental Data Table S1); gray points represent the replicates and are darker when points overlap; gray shading illustrates the 95% confidence bounds of the concentration‐response model (note: for total shoot dry weight the confidence bounds partly exceeded the display area); solid red dots represent EC50 estimates; EC50 estimates in each graph are given with lower and upper 95% confidence limits; NA indicates that an EC50 could not be reliably estimated. Note that *x*‐axes and *y*‐axes have different scales.

At 14‐d postexposure to glyphosate spray, *Ammannia* growth was inhibited at concentrations as low as 0.1% glyphosate (Figures 1 and  2). At concentrations of 0.1% and higher, glyphosate inhibited total root dry weight (ANOVA: *F*_6,35_ = 29.6; *p* < 0.001; Dunnett's posthoc: *p* < 0.001 for 0.1%, 0.36%, 1.34%, and 5% treatments) and *r*_*MSL*_ (ANOVA: *F*_6,35_ = 44.6; *p* < 0.001; Dunnett's posthoc: *p* < 0.001 for 0.1%, 0.36%, 1.34%, and 5% treatments). At concentrations of 0.36% and higher, glyphosate inhibited *r*_*TSL*_ (ANOVA: *F*_6,35_ = 31.8; *p* < 0.001; Dunnett's posthoc: *p* < 0.05 for 0.36% treatment, *p* < 0.001 for 1.34% and 5% treatments), and *r*_*SS*_ (ANOVA: *F*_6,35_ = 7.9; *p* < 0.001; Dunnett's posthoc: *p* < 0.05 for 0.36% treatment, *p* < 0.001 for 1.34% and 5% treatments). At concentrations of 1.34% and higher, glyphosate inhibited total shoot dry weight (ANOVA: *F*_6,35_ = 15.4; *p* < 0.001; Dunnett's posthoc: *p* < 0.001 for 1.34% and 5% treatments), and the total shoot dry matter content (ANOVA: *F*_6,35_ = 34.4; *p* < 0.001; Dunnett's posthoc: *p* < 0.01 for 1.34% treatment, *p* < 0.001 for 5% treatment). A comparison of EC50 values (estimate ± standard error) derived from concentration‐response modeling identified the most sensitive endpoint for *Ammannia* exposed to glyphosate spray, which was *r*_*MSL*_ (EC50 = 0.13 ± 0.05), followed by *r*_*TSL*_ (EC50 = 2.59 ± 0.36), and total shoot dry matter content (EC50 = 2.59 ± 0.36) (Figure 2). No reliable EC50 values were available for other assessment endpoints (Figure 2).

We detected low levels of glyphosate in soil collected from *Ammannia* sprayed with 0.1% to 5% glyphosate (maximum detection of 0.720 mg/kg fresh weight at 5%; equals 0.150 mg/kg dry weight), and detected AMPA in soil when plants were sprayed with 1.34% and 5% glyphosate (maximum detection of 0.022 mg/kg at 5%; equals 0.006 mg/kg dry weight) (Supplemental Data Figure S2).

### Experiment 2: *Sida* exposed to foliar glyphosate spray

**Figure 3:**
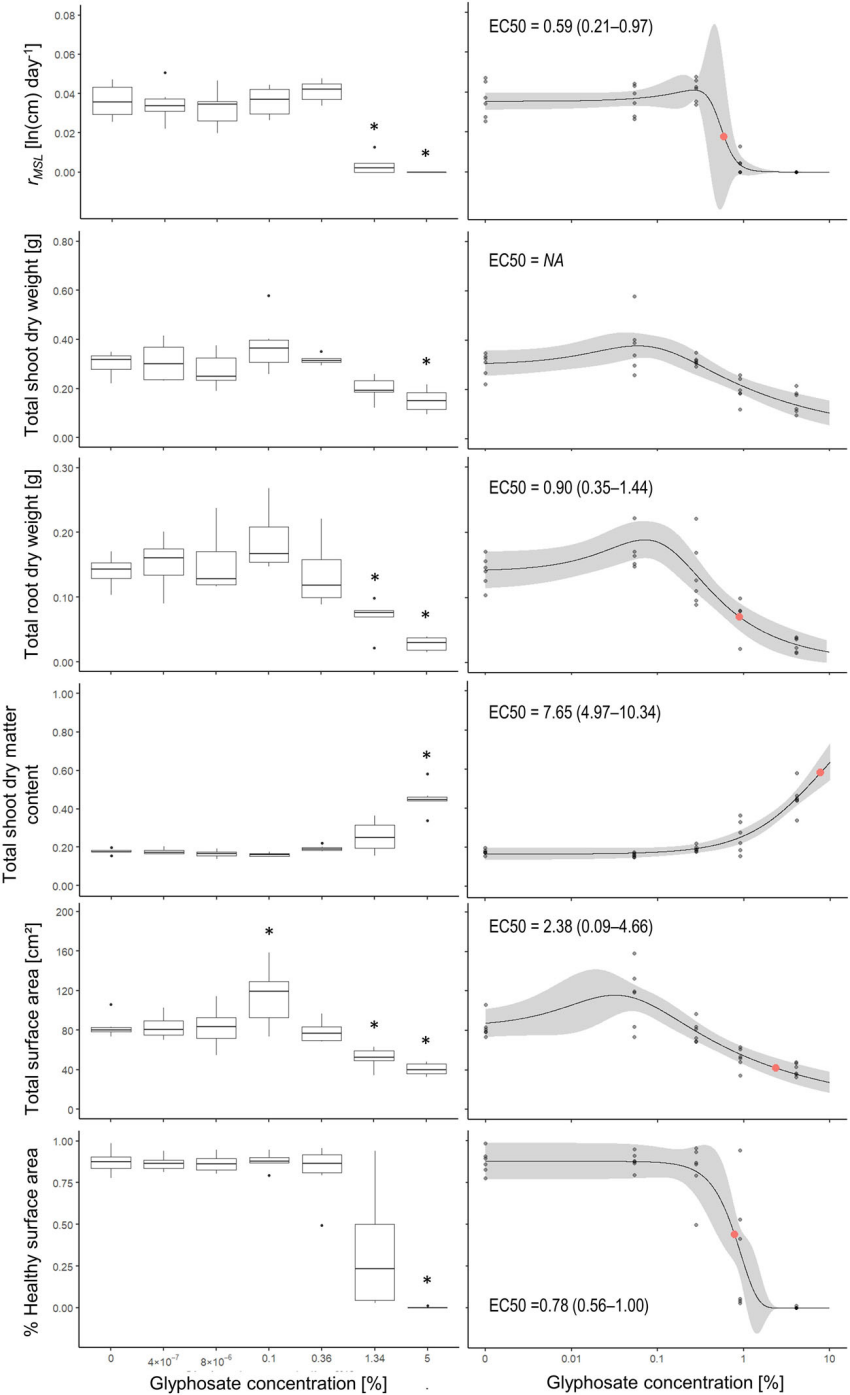
*Sida hermaphrodita* response to 0% to 5% (0–27 g/L) glyphosate spray 14‐d postexposure assessed based on 6 different endpoints (experiment 2). Each treatment was replicated 6 times. The left boxplot panels show results of an ANOVA analysis with Dunnett's posthoc test (*r*_*MSL*_, total shoot dry weight, total root dry weight, and total surface area) or Kruskal–Wallis analysis with Dunn's posthoc test (total shoot dry matter content, % healthy surface area) where “*” indicates a significant difference of a treatment to the control. The right panels show concentration‐response relationships for the same growth endpoints; measured concentrations were used for modeling (Supplemental Data Table S1); gray points represent the replicates and are darker when points overlap; gray shading illustrates the 95% confidence bounds of the concentration‐response model; solid red dots represent EC50 estimates; EC50 estimates in each graph are given with lower and upper 95% confidence limits; NA indicates that an EC50 could not be reliably estimated. Note that *x*‐axes and *y*‐axes have different scales.

At 14‐d postexposure to glyphosate spray, *Sida* growth was inhibited at concentrations as low as 1.34% glyphosate (Figures 1 and 3). *Sida* plants exposed to concentrations of 1.34% to 5% glyphosate exhibited decreased *r*_*MSL*_ (ANOVA: *F*_6,35_ = 32.4; *p* < 0.001; Dunnett's posthoc: *p* < 0.001 for 1.34% and 5% treatments) compared to control plants. Similarly, total root dry weight (ANOVA: *F*_6,35_ = 12.6; *p* < 0.001; Dunnett's posthoc: *p* < 0.05 for 1.34% treatment, *p* < 0.001 for 5% treatment) and total surface area (ANOVA: *F*_6,35_ = 12.4; *p* < 0.001; Dunnett's posthoc: *p* < 0.05 for 1.34% treatment, *p* < 0.001 for 5% treatment) were reduced in *Sida* plants sprayed with 1.34% to 5% glyphosate compared to the control. At 5% concentration, glyphosate spray inhibited total shoot dry weight (ANOVA: *F*_6,35_ = 7.7; *p* < 0.001; Dunnett's posthoc: *p* < 0.01 for 5% treatment) and total shoot dry matter content (Kruskal–Wallis: *χ* ^2^ = 26.4, *df* = 6, *p* < 0.001; Dunn's posthoc: *p* < 0.01 for 5% treatment). Likewise, the proportion of healthy surface area was inhibited by 5% glyphosate (Kruskal–Wallis: *χ* ^2^ = 20.5, *df* = 6, *p* < 0.01; Dunn's posthoc: *p* < 0.01 for 5% treatment). Conversely, 0.1% glyphosate spray positively affected total surface area of *Sida* plants (ANOVA: *F*_6,35_ = 12.4; *p* < 0.001; Dunnett's posthoc: *p* < 0.05 for 0.1% treatment).

A comparison of EC50 values (estimate ± standard error) derived from concentration‐response modeling identified *r*_*MSL*_ (EC50 = 0.59 ± 0.19) as the most sensitive endpoint for *Sida* exposed to glyphosate spray. The second most sensitive endpoint was proportion of healthy surface area (EC50 = 0.78 ± 0.11), followed by total root dry weight (EC50 = 0.90 ± 0.27), total surface area (EC50 = 2.38 ± 1.11), and finally total shoot dry matter content (EC50 = 7.65 ± 1.31) (Figure 3). No reliable EC50 was available for total shoot dry weight (Figure 3). Maximum hormesis obtained from concentration‐response modeling was 137% for the endpoint total surface area, 134% for total root dry weight, 125% for total shoot dry weight, and 115% for *r*_*MSL*_, relative to the control, which was considered to be 100%.

**Figure 4:**
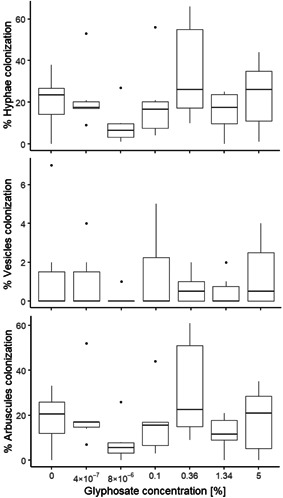
Mycorrhizal colonization of *Sida hermaphrodita* roots 14‐d postexposure to 0% to 5% (0–27 g/L) glyphosate spray, evaluated as % Hyphae, % Vesicles, and % Arbuscules colonization (experiment 2). No differences of mycorrhizal colonization were found between treatments and the control based on ANOVA analysis. Note that *y*‐axes have different scales.

Mycorrhizal colonization of *Sida* roots was not affected by glyphosate spray on the plants (Figure 4) when assessed as either hyphae (ANOVA: *F*_6,35_ = 1.4; *p* > 0.05), vesicles (ANOVA: *F*_6,35_ = 0.5; *p* > 0.05), or arbuscules (ANOVA: *F*_6,35_ = 1.5; *p* > 0.05).

We detected low levels of glyphosate in soil collected from *Sida* sprayed with 0.36% to 5% glyphosate (maximum detection of 0.160 mg/kg fresh weight at 5%; equals 0.070 mg/kg dry weight), and detected AMPA in soil when plants were sprayed with 1.34% and 5% glyphosate (detected, but below the limit of 0.005 mg/kg fresh weight) (Supplemental Data Figure S2).

### Experiment 3: *Ammannia* exposed to glyphosate in surface water

**Figure 5:**
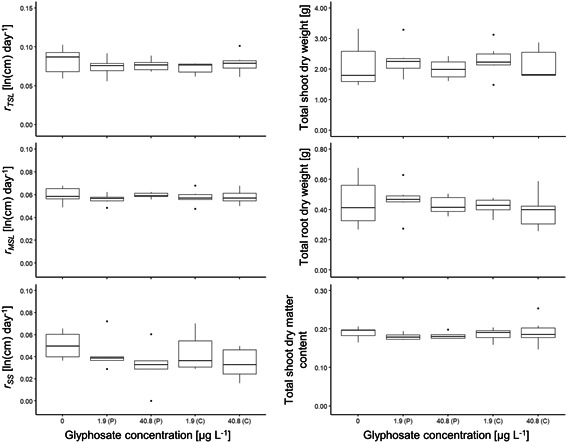
*Ammannia robusta* response to pulse (P) and continuous (C) low‐level glyphosate concentrations (water residues) of 1.9 and 40.8 µg/L (nominal treatments; see Supplemental Data Table S1 for measured concentrations) assessed 14‐d postexposure (continuous treatments were topped up daily during the 14 d) based on 6 different endpoints (experiment 3). Each glyphosate treatment was replicated 6 times, except the control treatment (0; *n* = 5). The ANOVA analysis with Dunnett's posthoc test resulted in no significant difference of any treatment to the control. Note that *x*‐axes and *y*‐axes have different scales.

Low‐level pulse or continuous exposures to glyphosate in water did not affect growth of *Ammannia* plants after 14 d (Figure 5). We detected no significant differences between any treatments and the control for all assessed endpoints, which included *r*_*TSL*_ (ANOVA: *F*_4,24_ = 0.5; *p* > 0.05), *r*_*MSL*_ (ANOVA: *F*_4,24_ = 0.3; *p* > 0.05), *r*_*SS*_ (ANOVA: *F*_4,24_ = 1.3; *p* > 0.05), total shoot dry weight (ANOVA: *F*_4,24_ = 0.3; *p* > 0.05), total root dry weight (ANOVA: *F*_4,24_ = 0.4; *p* > 0.05), and total shoot dry matter content, a proxy for moisture content (ANOVA: *F*_4,24_ = 0.4; *p* > 0.05).

We detected low levels of glyphosate in plant tissues at a maximum of 0.090 mg/kg fresh weight (=0.014 mg/kg dry weight) in the continuous treatment of 1.9 µg/L (Supplemental Data Figure S3). However, glyphosate was also detected at low levels of 0.034 mg/kg fresh weight (= 0.006 mg/kg dry weight) in the control treatment (note: the quantification limit was 0.030 mg/kg fresh weight). The glyphosate degradation product AMPA was not detected in plant tissues (Supplemental Data Figure S3). Glyphosate concentrations in the continuous exposure treatments did not decrease in the 24‐h period between daily top‐ups in the experimental trays (Supplemental Data Table S3).

### Experiment 4: *Ammannia* growing next to a glyphosate‐wicked invasive plant

**Figure 6:**
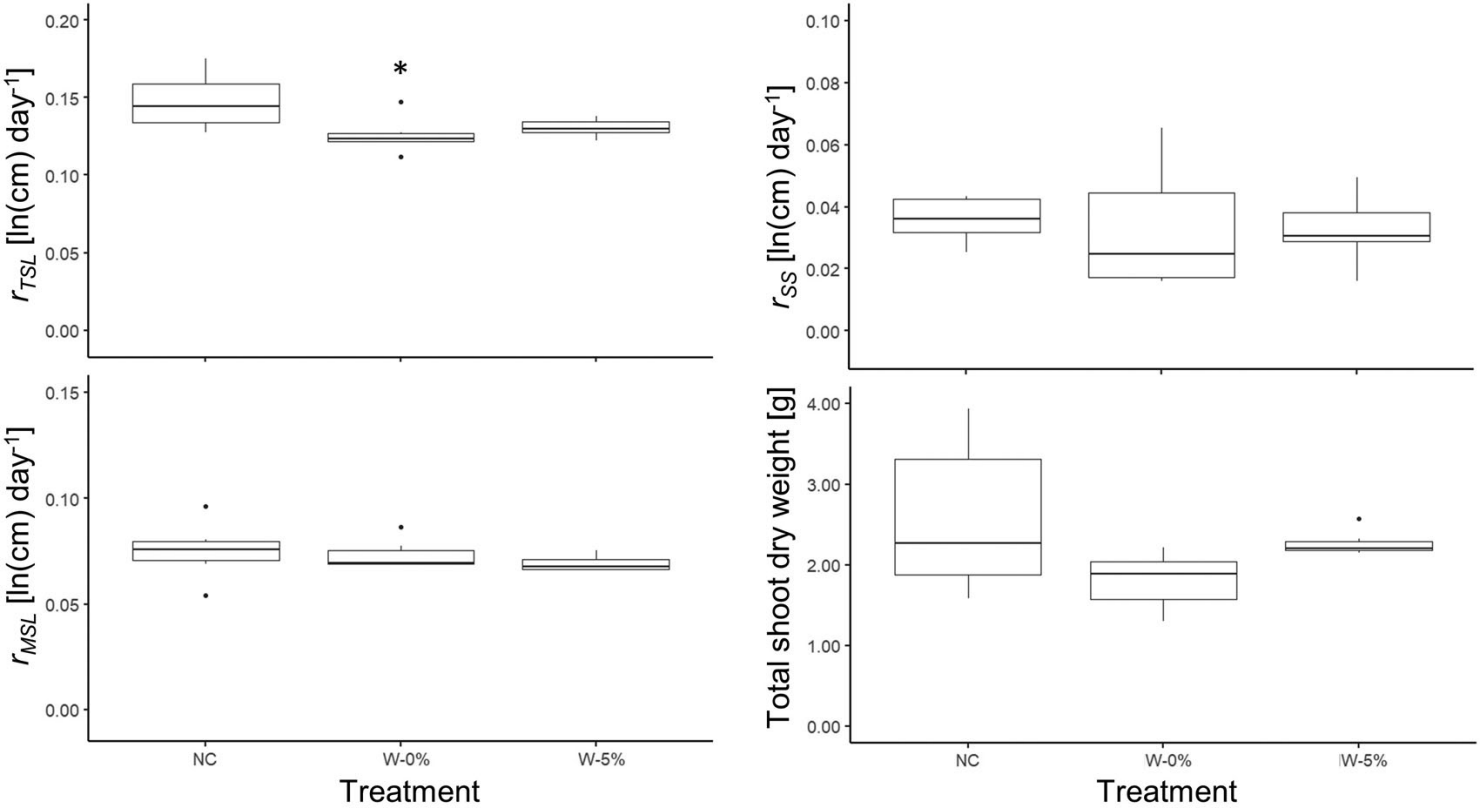
*Ammannia robusta* response 14 d after wicking of an adjacent *Typha* plant with 5% (27 g/L) glyphosate (W‐5%) or 0% glyphosate (control‐wicked plant; W‐0%) compared to a singly grown control plant (NC), based on 4 different endpoints (experiment 4). Each treatment was replicated 6 times. “*” indicates a significant difference between a treatment and NC based on an ANOVA analysis with Dunnett's posthoc test.

Wicking of adjacent plants with 5% glyphosate (W‐5%) did not negatively affect *Ammannia* growth compared to a singly grown control *Ammannia* plant (NC) (Figure 6). We detected no significant differences for our endpoints *r*_*TSL*_ (ANOVA: *F*_2,15_ = 4.6; *p* < 0.05; Dunnett's posthoc: *p* > 0.05 for HW‐5% treatment), *r*_*MSL*_ (ANOVA: *F*_2,15_ = 0.7; *p* > 0.05), *r*_*SS*_ (ANOVA: *F*_2,15_ = 0.1; *p* > 0.05), and total shoot dry weight (ANOVA: *F*_2,15_ = 2.5; *p* > 0.05). In contrast, neighboring invasive plants that were not wicked with glyphosate (W‐0%) negatively affected *Ammannia* growth compared to singly grown plants (NC), when assessed by the endpoint *r*_*TSL*_ (ANOVA: *F*_2,15_ = 4.6; *p* < 0.05; Dunnett's posthoc: *p* < 0.05 for W‐0% treatment) (Figure 6). However, this effect was not detected in the other 3 assessment endpoints (Figure 6).

## DISCUSSION

Our study assessed the response of nontarget, endangered macrophytes to glyphosate exposures that mimicked realistic scenarios encountered during invasive plant control. Exposure in native macrophytes was simulated using 3 pathways: interception of spray drift from nearby foliar spray applications; exposure to pulse or continuous glyphosate residues in water; and potential exposure while growing adjacent to invasive plants that receive glyphosate treatment via wicking of their leaves. At 14‐d postexposure, glyphosate spray reduced native plant growth at concentrations as low as 0.1%, and plants sprayed with 1.34% and 5% glyphosate showed almost complete decay. *Ammannia* was more sensitive overall to glyphosate spray than *Sida*, although sensitivity varied among measured endpoints. Conversely, low‐level pulse and continuous glyphosate residues in water did not affect *Ammannia* growth and survival. Likewise, *Ammannia* with rhizosphere contact to a glyphosate‐wicked invasive plant grew as well as *Ammannia* without neighbors. Our study is the first to compare glyphosate sensitivities among native, endangered emergent macrophyte species based on several growth endpoints, and to quantify responses to exposures mimicking multiple realistic pathways. Our study can therefore inform the development of glyphosate‐based invasive plant management practices that minimize unintended impacts on nontarget native macrophytes.

### Macrophyte sensitivity varies among measured endpoints

Ecological risk assessments of herbicides including glyphosate are performed in order to understand their potential threats to nontarget native biota. Glyphosate toxicity to nontarget macrophytes is typically assessed by comparing growth and biomass of treated versus untreated plants after a specific exposure period (commonly 14 d; OECD 2014). However, nontarget plant sensitivities vary depending on which aspects of growth and biomass are quantified: within 1 plant, EC50 estimates (as measures of sensitivity) based on different root and shoot growth and biomass endpoints can differ by factors of 10 to 1000 depending on the chemical tested (Arts et al. 2008). Our study assessed glyphosate sensitivity of *Ammannia* and *Sida* based on an array of 6 endpoints for each species, and our estimated EC50 values based on these endpoints varied by factors of 20 and 13, respectively. *Ammannia* (EC50 = 0.13%–2.59%) was more sensitive overall to glyphosate spray than *Sida* (EC50 = 0.59%–7.65%); however, the ranges of EC50 values overlap for both species, and therefore different conclusions may have been drawn if only a single endpoint at the lower or higher ends of the ranges had been measured for either species. While Arts et al. (2008) found that shoot endpoints (except the length of new shoots) were generally less sensitive than root endpoints, we detected no clear differences between shoot and root endpoints (Figures 2 and 3). Based on EC50 values, main shoot length (*r*_*MSL*_) was the most sensitive endpoint for both species in the present paper, although we were not able to estimate EC50 for all measured endpoints.

Ideally, macrophyte toxicity tests should include multiple endpoints (Arts et al. 2008; Maltby et al. 2010), but this may not be economically or technically feasible; hence, for each plant species, it is important to identify easy‐to‐measure endpoints that combine toxicological sensitivity and ecological relevance (Arts et al. 2008; Knauer et al. 2008), and from which concentration‐response curves can be obtained (Maltby et al. 2010). Applying these criteria to our results, we recommend that main shoot length be considered for assessing *Ammannia* and *Sida* responses to herbicides applied via foliar spray. Growth of the main shoot (i.e., endpoint “main shoot length”) is easy and noninvasive to measure, can be continuously assessed, and was toxicologically sensitive to glyphosate in both species. Root endpoints were also highly sensitive to glyphosate, but they could not be continuously measured in our test system because we grew plants in soil which would have required us to repeatedly excavate plants and clean roots prior to measurement; this is labor‐intensive and could impact plant health. In our study, reliable EC50 values based on root measurements could not be estimated for *Ammannia*. Future studies across emergent macrophyte taxa and different herbicides will be needed to determine whether main shoot length is a consistently sensitive endpoint that can be used as reference in future standardized guidelines for emergent macrophyte toxicity testing in pesticide risk assessment (as advocated for by the SETAC Europe workshop on Aquatic Macrophyte Risk Assessment for Pesticides) (Maltby et al. 2010).

### Glyphosate spray at low doses can stimulate growth in some macrophyte species

Most research concerning herbicides focuses on their toxic effects, but some chemicals, although toxic at higher doses, can be stimulatory at low doses (Duke et al. 2006). While glyphosate foliar spray at high concentrations can be toxic to plants, low concentrations can stimulate growth of many plant species; these contrasting effects (i.e., biphasic concentration‐response curve with inhibitory and stimulatory effects) are collectively referred to as “hormesis” (Brito et al. 2018). In our study, *Sida* exhibited a stimulus of 115% to 137% at low glyphosate doses compared to the control (100%) in 4 of our 6 measured endpoints (i.e., main shoot length, total shoot dry weight, total root dry weight, and total surface area). Our ANOVA analysis detected that the stimulatory effect was significant for the endpoint total surface area exposed to 0.1% glyphosate via foliar spray (Figure 3), but not for all other endpoints, possibly because the frequency and magnitude of stimulatory responses depend on the measured endpoint (Cedergreen et al. 2006). Since the total surface area is a proxy for the relative dose of glyphosate received (the larger the surface area, the more glyphosate can be intercepted), the inhibitory and stimulatory phases of its associated concentration‐response curve are likely more pronounced than in other endpoints. Conversely, *Ammannia* did not exhibit any detectable hormetic effect in response to glyphosate exposure. The different responses of the 2 species are plausible because the dose interval that promotes the stimulus can vary between species (Velini et al. 2008; Ather Nadeem et al. 2017; Brito et al. 2018). There are at least 3 potential explanations for our discrepant findings between species: *Ammannia* may experience a stimulatory effect at concentrations lower than our applied dose range; *Ammannia* plants may have been too young to experience a stimulatory effect, as this effect can vary among growth stages within 1 species (Velini et al. 2008; de Carvalho et al. 2013); or *Ammannia* simply does not experience a stimulatory effect in response to glyphosate exposure. Regardless, the underlying mechanisms of how glyphosate causes hormesis in plants are not clear (Velini et al. 2008; Brito et al. 2018). Since the dose ranges causing stimulatory effects are often narrow (Brito et al. 2018), it is unlikely that under “real‐world field conditions” nontarget endangered macrophytes will experience stimulatory effects from unintended glyphosate spray drift from nearby applications on invasive plants.

### Mycorrhizal fungi in macrophyte roots were not affected by glyphosate spray

Many macrophytes rely on associations between their roots and AMF to acquire resources (Smith and Read 2008; Stevens et al. 2011). Given this symbiotic relationship, the responses of AMF to herbicides can influence the host plant's herbicide tolerance (Ronco et al. 2008; Lekberg et al. 2017) and thereby its health. In our study, glyphosate foliar spray did not affect the abundance of AMF associated with *Sida*, even in treatments that produced low‐level glyphosate residues in the rhizosphere (0.36%–5% glyphosate treatments; see Supplemental Data Figure S2). Several earlier studies reported interactive effects between glyphosate and mycorrhizal fungi (Estok et al. 1989; Chakravarty and Chatarpaul 1990; Ronco et al. 2008), although these used either in vitro mycorrhizal growth tests without plants (Estok et al. 1989; Chakravarty and Chatarpaul 1990), or tests with plants exposed to glyphosate mixed into the soil they were growing in (Ronco et al. 2008). Similar to our findings, Lekberg et al. (2017) found no effects on AMF colonization of plant roots when another herbicide, picloram, was applied on plant foliage. However, the authors of that study also reported that a subsequently grown plant exhibited reduced mycorrhizal fungi colonization, suggesting that herbicide use can have indirect effects on mycorrhizal associations through plant community shifts to species with lower mycorrhizal dependency. Our study did not investigate mycorrhizal abundance in subsequently colonizing plants. Glyphosate exhibits lower persistence in environmental media compared to picloram (Lewis et al. 2016); therefore, exposure of recolonizing plants to glyphosate residues may be limited. However, given the current lack of knowledge, the potential longer‐term effects of glyphosate use on the abundance and colonization ability of mycorrhizal‐dependent endangered plants remain unclear.

### Exposure pathway can influence glyphosate toxicity to macrophytes

Glyphosate toxicity to macrophytes can vary with the exposure pathway. Lockhart et al. (1989) report that glyphosate spray resulted in high mortality of the surface‐floating *Lemna minor* L. despite it being relatively insensitive to glyphosate concentrations in the water, concluding that spray application deposit contact may pose a greater risk to emergent macrophytes than water contamination. Similarly, we found that *Ammannia* and *Sida* plants exposed to glyphosate spray exhibited reduced shoot and root growth at concentrations as low as 0.1% glyphosate, which is a small fraction (2%) of the 5% glyphosate spray that managers often apply to invasive *Phragmites* (Howell 2017; Tozer and Mackenzie 2019). However, we detected no effects on our nontarget macrophytes from adjacent wick‐applications with 5% glyphosate, or low‐level pulse and continuous water residues of 2 to 41 µg/L comparable to those previously detected in Ontario surface waters (CM Davy, Ontario Ministry of Natural Resources and Forestry, personal communication; Struger et al. 2008). This may be explained by the fact that different exposure pathways result in different amounts of glyphosate reaching a plant, which can in turn influence toxicity. Glyphosate spray directly exposes aerial plant tissues, and the larger the exposed surface area, the more glyphosate can be absorbed and potentially cause toxic effects (Cedergreen et al. 2004). Conversely, glyphosate in water likely has little tendency to partition to plants (Lockhart et al. 1989) due to its high affinity for soil particles (Saunders and Pezeshki 2015). In fact, we detected only minimal uptake of glyphosate from water into *Ammannia* shoot tissues (at concentrations near the quantification limit; 0.030 mg/kg fresh weight).

Given that glyphosate toxicity to macrophytes can vary with the exposure pathway, outcomes of glyphosate toxicity assessments may be influenced by the exposure pathway selected for the investigation. In most ecological risk assessments for pesticides in North America and the European Union, a substance's toxicity to nontarget macrophytes is initially assessed through aqueous exposure (water residues) tests (Maltby et al. 2010; EFSA PPR Panel 2013; USEPA 2017), and higher‐tier (more complex) assessments are triggered only if a pesticide “fails” the initial assessments due to unacceptable toxic effects (Maltby et al. 2010). However, spray drift exposure may identify glyphosate toxicity for emergent macrophytes that would not be detected in aqueous exposure tests and may therefore be more likely to lead to higher‐tier assessments. Therefore, we suggest that risk assessment procedures for nontarget macrophytes could add spray exposure tests with emergent species to their repertoire. Standard testing protocols for these tests could be adapted from existing guidelines for terrestrial nontarget plants (OECD 2006; USEPA 2012a, 2012b).

## CONCLUSION

Common goals of restoration are the removal of invasive plants, and the persistence or reestablishment of native plant communities (Hulme 2006; Flory and Clay 2009). Meeting both objectives should be more likely if glyphosate application methods are used that control invasive plants while posing minimum risks to adjacent nontarget plants. We recommend that glyphosate‐based invasive plant management near native and endangered macrophytes implements measures to limit off‐target spray drift and considers the feasibility of more targeted applications, such as with wipe equipment, a paintbrush, or squirt bottle. Further studies could identify the minimum effective glyphosate rates for invasive plants using these application methods. Control of invasive plants such as *Phragmites* should reduce their negative impacts on adjacent endangered plants; at the same time, selecting cautious and targeted application methods near any remaining endangered plants could lead to more successful conservation outcomes. Thus, implementing these recommendations into best management practices for invasive plants can support the development of effective restoration strategies for native plant communities in invaded aquatic and semiaquatic habitats.

## Disclaimer

The funders had no role in study design, data collection and analysis, decision to publish, or preparation of the manuscript. The views expressed in the publication are the views of the Government of Ontario grant recipients and do not necessarily reflect those of the Province.

## SUPPLEMENTAL DATA

The Supplemental Data contains several supplemental figues, tables, and texts, referred to in the main text. Supplemental figures display glyphosate residues in soil and plant tissues. Supplemental tables contain measured concentrations for the applied glyphosate treatments, and details on fitted concentration‐response models. Supplemental texts describe growth‐related assessments for Ammannia and Sida, the analysis of arbuscular mycorrhizal fungi in Sida roots, and the glyphosate and AMPA analysis performed at the Agriculture and Food Laboratory of University of Guelph.

## Supporting information

This article contains online‐only Supplemental Data.

Supporting information.

## Data Availability

Data are available on figshare at https://doi.org/10.6084/m9.figshare.12768053.v1.
