# Supplementary material for: Glyphosate Toxicity to Native Nontarget Macrophytes Following Three Different Routes of Incidental Exposure
Source: Integr Environ Assess Manag. 2020 Nov 5;17(3):597–613. doi: 10.1002/ieam.4350 (PMC8246556; doi:10.1002/ieam.4350)
Supplement: Supplementary file 1 — Supporting information. [file IEAM-17-597-s001.pdf]

SUPPLEMENTAL DATA

Glyphosate toxicity to native non-target macrophytes following three different routes of incidental exposure

This file includes:

SI Figures: Figures S.1 to S.3 (Pages 2–3)

SI Tables: Tables S.1 to S.3 (Pages 4–5)

SI Texts: Texts S.1 to S.3 (Pages 6–9)

SI References (Page 10)

SI Figures

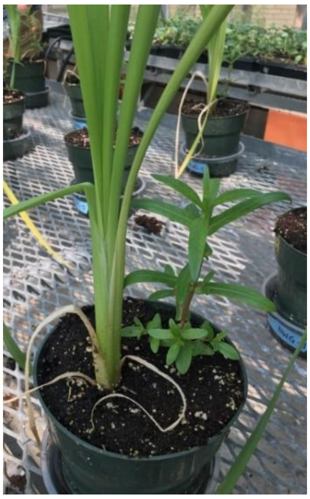

Figure S.1: Pots containing one *Typha* plant (left) adjacent to one *Ammannia* plant (right) so that both plants were spaced approximately 6 cm apart. We prepared 12 of these pots to be used in the experiment assessing effects from exposure to adjacent wicked plants via rhizosphere (Experiment 4).

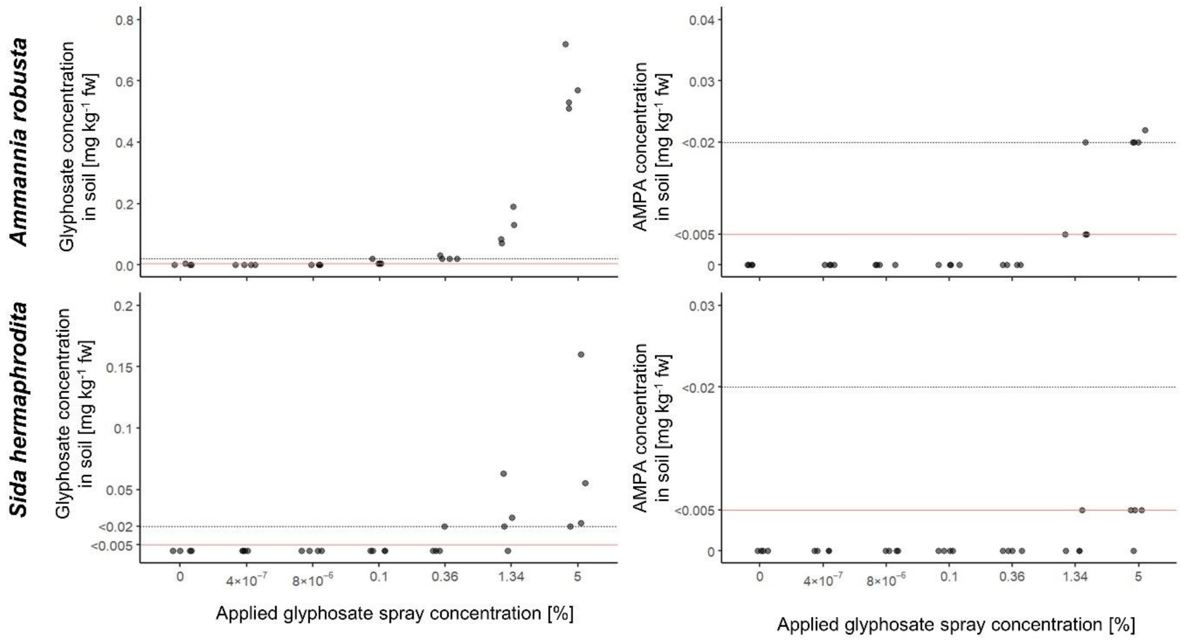

Figure S.2: Residues of glyphosate (left panels) and aminomethylphosphonic acid (AMPA; right panels) measured in rhizosphere soil of *Ammannia robusta* and *Sida hermaphrodita* at 14 days post-exposure to 0–5% glyphosate foliar spray (Experiments 1 and 2). Four replicate soil samples were analysed for each glyphosate treatment for both species at the Agriculture and Food Laboratory at University of Guelph (AFL; see Text S.3). Grey points represent the replicates. Black dashed lines represent the minimum quantification limit (0.02 mg kg^{-1} fresh weight) and red lines the minimum detection limit (0.005 mg kg^{-1} fresh weight) where AFL provided "smaller than" measurement values. Note y-axes have different scales. The proportion of dry matter of all samples ranged between 12–31% for *Ammannia*, and 39–55% for *Sida*.

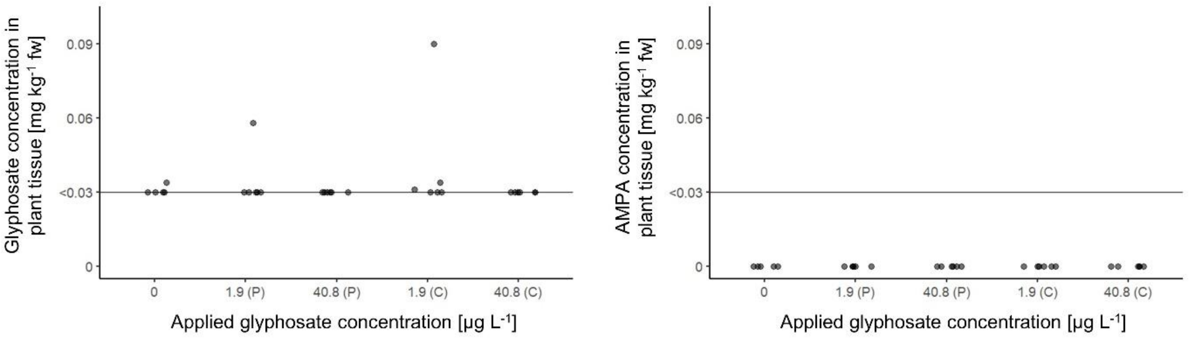

Figure S.3: Residues of glyphosate (left) and aminomethylphosphonic acid (AMPA; right) measured in shoot tissues of *Ammannia robusta* at 14 days post-exposure to pulse (P) and continuous (C) low level glyphosate water concentrations of 1.9 and 40.8 µg L^{-1} (nominal treatments; see table S.1 for measured concentrations) (Experiment 3). Each treatment was replicated six times, except the control treatment (0; n=5). Grey points represent the replicates. Plant tissues were analysed at the Agriculture and Food Laboratory at University of Guelph (AFL; see Text S.3). Black lines represent the minimum quantification limit (0.03 mg kg^{-1} fresh weight) where AFL provided “smaller than” measurement values. The proportion of dry matter of all samples ranged between 15–25%.

SI Tables

Table S.1: Nominal and measured concentrations for the applied glyphosate treatments (Roundup WeatherMAX® formulation); the concentrations of the active ingredient glyphosate were measured by the Agriculture and Food Laboratory at University of Guelph (AFL; see Text S.3); accuracy of applied treatments [%] was evaluated as ratio of measured (n = 1; results obtained from AFL) and nominal glyphosate concentrations; corresponding measured glyphosate treatments were calculated for further use in concentration-response analysis. In brackets, measurements of a second mix of the treatment solution are provided; a second mix was prepared for use in the continuous treatments in Experiment 3 from day 8 on. *ND* = Not detected (detection limit = 10^{-3} g L^{-1}); *NA* = Data not available;

Glyphosate treatment [%]	Corresponding nominal concentration [g L^{-1}]	Measured glyphosate concentration [g L^{-1}]	Accuracy [%] = (Measured/Nominal)×100	Corresponding measured glyphosate treatment [%]
5.00	27.0	22.00	81.48	4.07
1.34	7.2	4.90	67.72	0.91
0.36	1.9	1.50	77.16	0.28
0.10	0.5	0.29	53.70	0.05
$7.56 \cdot 10^{-6}$	$40.8 \cdot 10^{-6}$	$36 \cdot 10^{-6}$ ($70 \cdot 10^{-6}$)	88.24 (171.57)	$6.67 \cdot 10^{-6}$
$3.52 \cdot 10^{-7}$	$1.9 \cdot 10^{-6}$	*ND* ($29 \cdot 10^{-6}$)	*NA* (1526.32)	*NA*
0	0	0	100.00	0

Table S.2: Concentration-response models fitted to multiple assessment endpoints used to evaluate the effects of foliar glyphosate spray on *Ammannia* (Experiment 1) and *Sida* (Experiment 2). The chosen models represent best-fitting models as determined by using the "mselect" function in "drc" package in R, which compares models using the following criteria: log likelihood value, Akaike's information criterion (AIC), estimated residual standard error, and lack-of-fit test p-value (Ritz et al. 2015). The models were used for absolute EC50 and confidence interval estimation via the "ED" function

Macrophyte	Assessment endpoint	Concentration-response model fitted
Ammannia	r_{TSL}	Three-parameter Weibull function "W2.3"
	r_{MSL}	Three-parameter Weibull function "W1.3"
	r_{SS}	Three-parameter Weibull function "W1.3"
	Total shoot dry weight	Four-parameter Weibull function "W1.4"
	Total shoot dry matter content	Three-parameter log-logistic function with upper limit fixed at one "LL.3u"
	Total root dry weight	Three-parameter Weibull function "W1.3"
Sida	r_{MSL}	Brain-Cousens hormesis model function "BC.4"
	Total surface area	Brain-Cousens hormesis model function "BC.4"
	% Healthy surface area	Three-parameter Weibull function "W1.3"
	Total shoot dry weight	Brain-Cousens hormesis model function "BC.4"
	Total shoot dry matter content	Three-parameter log-logistic function with upper limit fixed at one "LL.3u"
	Total root dry weight	Brain-Cousens hormesis model function "BC.4"

Table S.3: Comparison of measured glyphosate concentrations in our mixed treatment solution (second mix; see Table S.1) versus concentrations detected in the water collected from continuous treatment trays in Experiment 3 at 24 hours after topping up; concentrations were measured by the Agriculture and Food Laboratory at University of Guelph (see Text S.3); change of glyphosate concentration in 24 hours was evaluated as ratio of measured in tray (n=4) versus measured in mixed treatment solution (n=1)

**Treatment solution concentration [μg L^{-1}]**	**Average (± standard error) concentration measured in continuous treatment trays at 24 hours after top-up [μg L^{-1}]**	**24-hour concentration change [%]**
70	74.0±1.5	106
29	31.3±0.7	108

SI Texts

Text S.1: Detailed descriptions of growth-related assessments for *Ammannia* and *Sida*

Several growth-related endpoints were measured to assess the sensitivity of *Ammannia* and *Sida* to glyphosate exposure. *Ammannia* and *Sida* differ in their morphology, which is why we used different endpoints for either species to assess their response. Details on how each endpoint was measured are below (with the respective plant species in brackets).

<u>Main shoot length (*Ammannia* and *Sida*)</u>

Main shoot length was measured with a ruler from soil level to the top of the main stem. These measurements were taken immediately prior to initiation of the experiment (day 0) and upon completion of the experiment (day 14). On day 14, some plants were dried out, shrunk and curled up (see in Fig. 1 at high glyphosate spray concentrations), which complicated accurate measurements of main shoot length as plants were starting to fall apart upon touching them. In these cases where no growth occurred over 14 days, main shoot length for day 14 was recorded as identical to day 0.

<u>Total shoot length (*Ammannia*)</u>

Total shoot length for a plant was measured as the combined length of main shoot and all side shoots of the individual plant (> 1 mm). With a ruler, the length of the main shoot was determined as described above, and the length of each side shoot was measured similarly starting from the branching point on the main shoot to the tip of the side shoot. All measurements were summed up to calculate the total shoot length. These measurements were taken immediately prior to initiation of the experiment (day 0) and upon completion of the experiment (day 14). On day 14, some plants were dried out, shrunk, and curled up (see in Fig. 1 at high glyphosate spray concentrations), which complicated accurate measurements of total shoot length as plants were starting to fall apart upon touching them. In these cases where no growth occurred over 14 days, total shoot length for day 14 was recorded as identical to day 0.

<u>Number of side shoots (*Ammannia*)</u>

Number of side shoots was counted as the number of shoots (> 1 mm) branching from the main stem. These measurements were taken immediately prior to initiation of the experiment (day 0) and upon completion of the experiment (day 14).

Total surface area (*Sida*)

Total surface area was determined by separating leaves from the stem for each plant, placing all parts flat on a scanner (EPSON Expression 10000XL 1.0), and using the software WinRHIZO™ 2012d (Regent Instruments Canada Inc. 2011) to estimate the total surface area of leaves and stem. These measurements were taken upon completion of the experiment (day 14) as plants needed to be harvested for it.

Proportion of (%) healthy surface area (*Sida*)

Based on our results from our analysis of total surface area, we used the software feature "colour analysis" in WinRHIZO™ 2012d (Regent Instruments Canada Inc. 2011) to determine the proportion of healthy surface area for each individual plant, defined as the proportion of all leaves and stem coloured in shades of green. These measurements were taken upon completion of the experiment (day 14) as plants needed to be harvested for it.

Total shoot dry weight (*Ammannia* and *Sida*)

To determine total shoot dry weight, in a first step each individual plant was cut at soil level, and the entire shoot was weighed to determine total shoot fresh weight (which is later used to determine the total shoot dry matter content). Subsequently, the entire shoot was placed in a brown paper bag into a drying oven at 70–90 °C for 48–120 hours until completely dry, and then re-weighed to determine total shoot dry weight. These measurements were taken upon completion of the experiment (day 14) as plants needed to be harvested for it.

As an exemption, for Experiment 3 where parts of the fresh shoot were needed for glyphosate analysis, a representative subsample of the entire fresh shoot was taken, weighed, and placed in a brown paper bag into a drying oven at 70-90 °C for 48-120 hours until completely dry, and then re-weighed to determine dry weight of the subsample. For each subsample, the proportion of dry matter was calculated by dividing its dry weight by its fresh weight. The subsample proportion of dry matter was then multiplied by the total shoot fresh weight, to calculate the total shoot dry weight for each individual.

Total shoot dry matter content (*Ammannia* and *Sida*)

The total shoot dry matter content was calculated by dividing total shoot dry weight by total shoot fresh weight for each individual plant. Total shoot dry weights and fresh weights were

measured as described above. These measurements were taken upon completion of the experiment (day 14) as plants needed to be harvested for it.

Total root dry weight (*Ammannia* and *Sida*)

To determine total root dry weight, in a first step each individual plant was cut at soil level, and the roots were separated from the soil and thoroughly rinsed under running tap water. Subsequently, all roots for each individual were placed in a brown paper bag into a drying oven at 70–90 °C for 48–120 hours until completely dry, and then re-weighed to determine total root dry weight. These measurements were taken upon completion of the experiment (day 14) as plants needed to be harvested for it.

As an exemption, for Experiment 2 where parts of the fresh roots were needed for mycorrhizal fungi analysis, a representative subsample of the fresh root system was taken, weighed, and placed in a brown paper bag into a drying oven at 70-90 °C for 48-120 hours until completely dry, and then re-weighed to determine dry weight of the subsample. For each subsample, the proportion of dry matter was calculated by dividing its dry weight by its fresh weight. The subsample proportion of dry matter was then multiplied by the total root fresh weight, to calculate the total root dry weight for each individual.

Text S.2: Analysis of arbuscular mycorrhizal fungi (AMF) in *Sida* roots (Experiment 2)

We analysed mycorrhizal colonization in *Sida* roots as the proportion of root cortical cells colonized by the three main structures of arbuscular mycorrhizal fungi (the most common mycorrhizal type; Smith and Read 2008): hyphae, vesicles, and arbuscules. For mycorrhizal analysis, the following steps were performed on subsamples of *Sida* roots from all glyphosate spray treatments (Vierheilig et al. 1998; Mulholland 2019): rinsing in deionized water to remove ethanol from storage; submerging in 10% potassium hydroxide (CAS 1310-58-3, Fisher Scientific, Janssen Pharmaceuticalaan 3a, 2440 Geel, Belgium); placing in a vacuum oven (Thermo Scientific Lindberg Blue M) at 95 °C and 20 in Hg pressure for 30 minutes; rinsing in 10% white vinegar solution (Great Value, Walmart Canada Corp., 1940 Argentia Rd, Mississauga, ON L5N 1P9); submerging in a solution of 5% ink (Sheaffer Skrip, MMIX Sheaffer Slovakia, Prienyselná 1, 926 01, Sered, Slovak Republic) and 95% white vinegar; placing in the vacuum oven at 95 °C and 20 inHg pressure for 30 minutes; rinsing in 10% white vinegar; and submerging in 50% glycerol solution (CAS 56-81-5; Fisher Scientific, 1 Reagent Lane, Fair Lawn, NJ 07410) for 20 hours for de-staining. Roots were then laid out in a glass bowl with flat bottom placed on a 5×5 grid, and root fractions were sampled randomly

from grid squares using a random number generator. Ten to twenty first-order fine roots were selected and mounted lined up on microscope slides (VWR Microscope Slide Frosted 75 × 25 × 1 mm; CAT 82027-132), wetted with 50% glycerol, and covered with microscope cover glass (Fisherbrand Microscope Cover Class 60 × 25 × 1 mm, 12-545M; LOT 18921; 300 Industry Drive, Pittsburgh, PA 15275). Colonization levels were assessed using the magnified intersections method (McGonigle et al. 1990). For every slide, 100 unique fields of view across all root fractions were assessed for presence or absence of the mycorrhizal structures "Hyphae", "Vesicles", and "Arbuscules" using a microscope (Nikon Eclipse E600) with 10×/0.30 magnification. From this, the proportion of hyphal, arbuscular, and vesicular AMF colonization was calculated by dividing the number of fields of view with mycorrhizal structures present by the total number of fields of view assessed (= 100).

Text S.3: Glyphosate and AMPA analysis performed at the Agriculture and Food Laboratory of University of Guelph

Several water, plant, and soil samples were submitted to the Agriculture and Food Laboratory of University of Guelph (AFL) for analysis of glyphosate and aminomethylphosphonic acid (AMPA; one common degradation product) concentrations. At AFL, all samples are homogenized so that the relatively small (5–100 g) test portions taken for analysis are representative of the entire sample. For all samples, the laboratory prepared an aqueous extract which was then acidified and separated from co-extractives using solid phase extraction. The samples were analysed using Liquid chromatography–mass spectrometry/Mass spectrometry (LC-MS/MS). The LC-MS/MS system employed a cation guard column (Micro-Guard Cation-H cartridge 30 X 4.6 mm) for chromatographic separation, a mobile phase A (0.1% formic acid in nanopure grade H_2O) and B (acetonitrile), with a flow rate of 1 mL min^{-1}, a total run time of 12 min, and retention times of 0.9 min for glyphosate and 4.2 min for AMPA. Auto-sampler temperature was set at 8 °C, and the injection volume was 50 µL. Column oven temperature was set at 20 ± 3 °C. Laboratory detection limits for glyphosate and AMPA were 0.005 mg kg^{-1} fresh weight in plant tissue and soil, and 0.001 mg L^{-1} in water; quantification limits were 0.03 mg kg^{-1} fresh weight in plant tissue, 0.02 mg kg^{-1} fresh weight in soil, and 0.008 mg L^{-1} in water. Recoveries were on average (± standard error) 78.1 ± 1.8% for glyphosate and 79.7 ± 1.9% for AMPA.

References

McGonigle TP, Miller MH, Evans, DG, Fairchild GL, Swan JA. 1990. A new method which gives an objective measure of colonization of roots by vesicular-arbuscular mycorrhizal fungi. In *New Phytol* 115 (3): 495–501. DOI: 10.1111/j.1469-8137.1990.tb00476.x.

Mulholland SN. 2019. Competition or facilitation: Examination of interactions between endangered *Sida hermaphrodita* and invasive *Phragmites australis*. Master of Science. Wilfrid Laurier University. Department of Biology. Available online at https://scholars.wlu.ca/etd/2223.

Regent Instruments Canada Inc. 2011. WinRHIZO 2012b: Basic, Reg, Pro & Arabidopsis For Root Measurement.

Ritz C, Baty F, Streibig JC, Gerhard D. 2015. Dose-response analysis Using R. In *PloS one* 10 (12): e0146021. DOI: 10.1371/journal.pone.0146021.

Smith SE, Read DJ. 2008. Mycorrhizal symbiosis. 3rd ed. Amsterdam, Boston: Elsevier/Academic Press.

Vierheilig H, Coughlan AP, Wyss U, Piche Y. 1998. Ink and vinegar, a simple staining technique for arbuscular-mycorrhizal fungi. In *Applied and Environmental Microbiology* 64 (12): 5004-5007. DOI: 10.1128/AEM.64.12.5004-5007.1998
